# NLRP3 regulates alveolar bone loss in ligature‐induced periodontitis by promoting osteoclastic differentiation

**DOI:** 10.1111/cpr.12973

**Published:** 2020-12-31

**Authors:** Yuyi Chen, Qiudong Yang, Chunhua Lv, Yue Chen, Wenhua Zhao, Wenlei Li, Hongyu Chen, Hua Wang, Wen Sun, Hua Yuan

**Affiliations:** ^1^ Jiangsu Key Laboratory of Oral Diseases Nanjing Medical University Nanjing China; ^2^ Department of Oral and Maxillofacial Surgery Affiliated Hospital of Stomatology Nanjing Medical University Nanjing China; ^3^ Department of Stomatology Taizhou People’s Hospital of Jiangsu Province Taizhou China; ^4^ Department of Basic Science of Stomatology Affiliated Hospital of Stomatology Nanjing Medical University Nanjing China

**Keywords:** alveolar bone loss, inflammasome, MCC950, NLRP3, osteoclast differentiation, periodontitis

## Abstract

**Objectives:**

NLRP3 inflammasome is a critical part of the innate immune system and plays an important role in a variety of inflammatory diseases. However, the effects of NLRP3 inflammasome on periodontitis have not been fully studied.

**Materials and methods:**

We used ligature‐induced periodontitis models of NLRP3 knockout mice (NLRP3^KO^) and their wildtype (WT) littermates to compare their alveolar bone phenotypes. We further used Lysm‐Cre/Rosa^nTnG^ mouse to trace the changes of Lysm‐Cre^+^ osteoclast precursors in ligature‐induced periodontitis with or without MCC950 treatment. At last, we explored MCC950 as a potential drug for the treatment of periodontitis in vivo and in vitro.

**Results:**

Here, we showed that the number of osteoclast precursors, osteoclast differentiation and alveolar bone loss were reduced in NLRP3^KO^ mice compared with WT littermates, by using ligature‐induced periodontitis model. Next, MCC950, a specific inhibitor of the NLRP3 inflammasome, was used to inhibit osteoclast precursors differentiation into osteoclast. Further, we used Lysm‐Cre/Rosa^nTnG^ mice to demonstrate that MCC950 decreases the number of Lysm‐Cre^+^ osteoclast precursors in ligature‐induced periodontitis. At last, treatment with MCC950 significantly suppressed alveolar bone loss with reduced IL‐1β activation and osteoclast differentiation in ligature‐induced periodontitis.

**Conclusion:**

Our findings reveal that NLRP3 regulates alveolar bone loss in ligature‐induced periodontitis by promoting osteoclastic differentiation.

## Graphical abstract

NLRP3 Regulates Alveolar Bone Loss in Ligature‐induced Periodontitis by Promoting Osteoclastic Differentiation. Bacterial infection in periodontal tissues leads to the activation of NLRP3 inflammasome in osteoclast precursor cells, which promotes osteoclast differentiation and alveolar bone resorption. Thus, NLRP3 inflammasome contribute significantly to the pathologic bone loss in periodontitis. MCC950, a specific inhibitor of the NLRP3 inflammasome, inhibits osteoclast differentiation, thereby reduces alveolar bone loss in periodontitis.

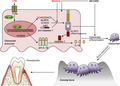

## 1 INTRODUCTION

Periodontitis is a chronic inflammatory and infectious disease affecting the periodontal tissues around the teeth, characterized by periodontal attachment loss, bone resorption, and finally leading to tooth loss.^1^ Periodontitis is one of the most common oral diseases, with a high incidence in adults.^2^ It is noteworthy that the inflammation and the spread of bacterial components during periodontitis have a close relationship with various systemic diseases, including Rheumatoid arthritis (RA), cardiovascular diseases and cancer.^3^

Inflammasomes are multi‐protein complexes assembled by intracytoplasmic pattern recognition receptors (PRRs), which are an important part of the body's immune system.^4^ Nucleotide binding oligomerization domain (Nod)‐like receptor family pyrin domain‐containing 3 (NLRP3) inflammasome is one of the most typical inflammasomes, which is composed of NLRP3, apoptosis‐associated speck‐like protein containing a caspase‐1 recruitment domain (ASC) and pro‐caspase‐1. Toll‐like receptors (TLRs) are a group of PRRs,^5^ which can recognize pathogen‐associated molecular patterns (PAMPs) and damage‐associated molecular patterns (DAMPs) and activate nuclear factor kappa B (NF‐κB), resulting the formation of pro‐IL‐1β and up‐regulated NLRP3 expression. This is the first step of NLRP3 inflammasome activation. The second step leads to the cleavage of pro‐caspase‐1 to caspase‐1, followed by the conversion of pro‐IL‐1β to IL‐1β through assembling NLRP3, ASC and pro‐caspase‐1 into an inflammasome complex.^6^, ^7^, ^8^ In recent years, NLRP3 inflammasome has attracted widespread attention due to its extensive function in inflammatory diseases.^9^ With the in‐depth research on the mechanism of NLRP3 inflammasomes, a variety of small molecule compounds have been found to act as inhibitors of NLRP3 inflammasome for the treatment of NLRP3‐mediated inflammatory diseases, such as MCC950, CY‐09, INF39.^10^, ^11^

Recently, more attention has been paid to the association between NLRP3 inflammasome and periodontitis. In a mouse periodontitis model, knock out of NLRP3 decreases Porphyromonas gingivalis‐associated alveolar bone loss.^12^ Consistent results also observed in human tissue, a research shows NLRP3 and IL‐1β have been found highly expressed in human gingival tissues with severe chronic periodontitis. NLRP3 expression is up‐regulated with the treatment of stimulus in many periodontal cell types, including macrophage, periodontal ligament fibroblasts (PDLFs) and periodontal ligament cells (PDLCs).^13^, ^14^ However, it is well known that NLRP3 is highly expressed in monocytes/macrophages,^15^ which could further differentiate into osteoclasts under suitable conditions. Therefore, we intend to investigate whether NLRP3 can regulate periodontitis‐related alveolar bone loss by affecting osteoclast differentiation.

In this study, we used ligature‐induced periodontitis models of NLRP3 knockout mice (NLRP3^KO^) and their wildtype (WT) littermates to compare their alveolar bone phenotypes. We further used Lysm‐Cre/Rosa^nTnG^ mouse to trace the changes of Lysm‐Cre^+^ osteoclast precursors in ligature‐induced periodontitis with or without MCC950 treatment. At last, we explored MCC950 as a potential drug for the treatment of periodontitis in vivo and in vitro.

## 2 MATERIALS AND METHODS

### 2.1 Animals and Ligature‐induced periodontitis

NLRP3^KO^ mice generated in a C57BL/6J background were kindly provided by Professor Shuo Yang.^16^ The LysM‐Cre mouse line was obtained from Gem Pharmatech Company (Stock Number T003822) and the Rosa^nTnG^ mouse line was obtained from the Jackson Laboratory (Stock Number 023035). Lysm‐Cre/Rosa^nTnG^ mice were obtained by crossing Lysm‐Cre mice with Rosa^nTnG^ mice. Experimental periodontitis was induced in 2‐month‐old WT, NLRP3^KO^ and Lysm‐Cre/Rosa^nTnG^ mice. Briefly, 5‐0 silk ligature was tied around the right maxillary second molar and the other side was left untied to serve as the baseline control. MCC950 (MCE, Cat#HY‐12815) or PBS was injected intraperitoneally (i.p) to mice (10 mg/kg) at day 0, 1 and 2 and every 2 days thereafter. Mice were sacrificed 10 days after placement of the ligature. All mice were bred and maintained in the SPF Laboratory Animal Center of Nanjing Medical University. All animal procedures were conducted in accordance with approved guidelines of the Committee of Nanjing Medical University for Animal Resources (Approval ID 1906018).

### 2.2 Micro‐computed tomography (micro‐CT)

Maxillae were removed and dissected free of all soft tissues for micro‐computed tomography (Micro‐CT) as described.^17^

### 2.3 Histology and histochemistry staining

Isolated maxillae were fixed in 4% paraformaldehyde, and then decalcified in 14% EDTA. After dehydration, the maxillae were embedded in paraffin for paraffin sections or embedded in Tissue‐Tek OCT compound for frozen sections. The paraffin sections were stained with haematoxylin and eosin (H&E), histochemically for tartrate‐resistant acid phosphatase (TRAP) or alkaline phosphatase (ALP) and analysed as described previously.^18^ The frozen sections of Lysm‐Cre/Rosa^nTnG^ mice were mounted with Mounting Medium containing DAPI (Vector) and images were captured with a Leica DM4000 fluorescence microscope, as we reported previously.^19^

### 2.4 Immunohistochemistry staining

The deparaffinized sections were subjected to heat mediated antigen retrieval, and blocked in H_2_O_2_ for 30 minutes, followed by PBS with 5% BSA and 0.2% Triton X‐100 at room temperature for 30 minutes, and then stained overnight with primary antibody against IL‐1β (R&D System, Cat#AF‐401‐NA). After rinsing with PBS for 15 minutes, tissues were incubated with secondary antibody (HRP‐Donkey Anti‐Goat IgG, Proteintech, Cat#SA00001‐3) at room temperature. Sections were then washed and colours were developed with DAB (3,3’‐diaminobenzidine). Next, haematoxylin was used as a counterstain. Images were captured with a Leica DM4000 fluorescence microscope.

### 2.5 Flow cytometry

For flow cytometric analysis, cells were harvested from periodontal tissues around maxillary second molar as described previously.^18^ Single cell suspensions were stained with fluorochrome‐conjugated antibodies against CD45.2 (eBioscience, Cat#47‐0454‐82), F4/80 (eBioscience, Cat#45‐4801‐82), CD11b (eBioscience, Cat#12‐0112‐85) and Gr‐1(eBioscience, Cat#25‐5931‐81). Flow cytometry was performed using an 8‐colour LSRII (eBioscience). Results were analysed by Flowjo7 data analysis software. For flow cytometric sorting, bone marrow (BM) cells were collected from femora and tibiae of Lysm‐Cre/Rosa^nTnG^ mice. Flow cytometry was performed using an 11‐colour LSRII to sort out Lysm‐Cre‐GFP^+^ tdTomato^−^ cells. Lysm‐Cre‐GFP^+^ tdTomato^−^ cells were further subjected to osteoclastogenic assays or real‐time PCR as follow.

### 2.6 Cell culture and Inflammasome activation

Tibiae and femora were obtained and BM cells were flushed out using a‐MEM/2% FBS. After red blood cells were lysed, BM cells were cultured in α‐MEM supplemented with 10% foetal Bovine serum (FBS), 1% penicillin/streptomycin (P/S) and 25 ng/mL M‐CSF for 3 days to generate osteoclast precursors (OCPs). OCPs were primed with 100 ng/mL LPS for 3 hours in serum free medium, and then treated with MCC950 (0.1 µmol/L) for 30 minutes before being stimulated with Nigericin (10 μmol/L) for 30 minutes. Cell lysates and supernatants were collected to detect NLRP3 inflammasome activation via Western blot and ELISA. For osteoclastogenic assays, BM cells were harvested and cultured in 96‐well plates (5 × 10^4^ cells/well) in α‐MEM with 10% FBS, 1% penicillin/streptomycin (P/S) containing 25 ng/mL M‐CSF and 10 ng/mL RANKL for 4 days. On day 5, stimulus were used to activate inflammasome as above. Then cells were fixed by 4% paraformaldehyde and stained for TRAP activity. TRAP‐positive cells with more than 3 nuclei were counted for TRAP^+^ osteoclast number by using inverted microscopy. For blocking experiments with neutralizing Ab, anti‐mouse IL‐1β Ab (R&D System, Cat#AF‐401‐NA) was purchased from R & D systems and used according to the manufacturer's instructions.

### 2.7 ELISA

Cell supernatants were removed and analysed using ELISA kits according to the manufacturer's instructions to measure the release of IL‐1β (Invitrogen, Cat#88‐7013).

### 2.8 Western blot analysis

The protein content of supernatants was concentrated using Amicon^®^ Ultra‐4 Centrifugal Filter Unit (Merck Millipore, Cat#UFC8010) according to the manufacturer's instructions. Meanwhile, cell lysates were lysed directly in RIPA buffer. Protein samples were quantitated by a BCA protein assay kit (Beyotime, Cat#P0012) and then separated by SDS‐PAGE and transferred onto polyvinylidene diflouride (PVDF) membrane. Membranes were blocked in 5% milk in PBS for 3 hours at room temperature. After that, membranes were incubated at 4°C overnight with primary antibodies against NLRP3 (Cell Signaling Technology, Cat#D4D8T), Caspase‐1 (AdipoGen, Cat#AG‐20B‐0042), IL‐1β (R&D System, Cat#AF‐401‐NA) and β‐actin (Santa Cruz, Cat#sc‐47778). The next day, after washing with PBST for 3 times, each for 10 minutes, membranes were incubated with second antibodies for 1 hour. Specific bands were developed using enhanced chemiluminescence (ECL) reagents (Tanon), visualized by Tanon‐5200 Multi Chemiluminescent System (Tanon).

### 2.9 Immunocytochemistry

Cells were fixed in 4% paraformaldehyde before being blocked in PBS with 10% normal goat serum and 0.1% Triton X‐100 for 30 minutes, and then stained overnight with Goat anti‐IL‐1β (R&D System, Cat#AF‐401‐NA) or Goat anti‐NLRP3 (Abcam, Cat#ab4207)at 4°C. After washing with PBS for 30 minutes, cells were incubated respectively with Donkey anti‐Goat Cy3 (Beyotime, Cat#A0502) at room temperature. Cells were stained with DAPI and images were captured with a Leica DM4000 fluorescence microscope.

### 2.10 Quantitative real‐time PCR

Total RNA of osteoclasts was extracted with TRIzol Reagent (Invitrogen, Cat#15596026). cDNAs were reversely transcribed with the PrimeScript RT Master Mix (Takara, Cat#RR036A) and subjected for RT‐qPCR using specific primers. The sequences of the forward and reverse primers are listed in Supplementary Table S1 on line.

### 2.11 Statistical analysis

All data are given as mean ± SD. Statistical analysis was performed using graphpad prism 7 software (GraphPad Software Inc, San Diego, CA, USA). Comparisons between 2 groups were analysed using the 2‐tailed unpaired Student's *t*‐test. Comparisons among 3 or more groups were carried out using one‐way ANOVA followed by Dunnett's post‐hoc multiple comparisons. *P* values <.05 were considered statistically significant.

## 3 RESULTS

### 3.1 NLRP3 deficiency protects against alveolar bone loss in ligature‐induced periodontitis

**FIGURE 1:**
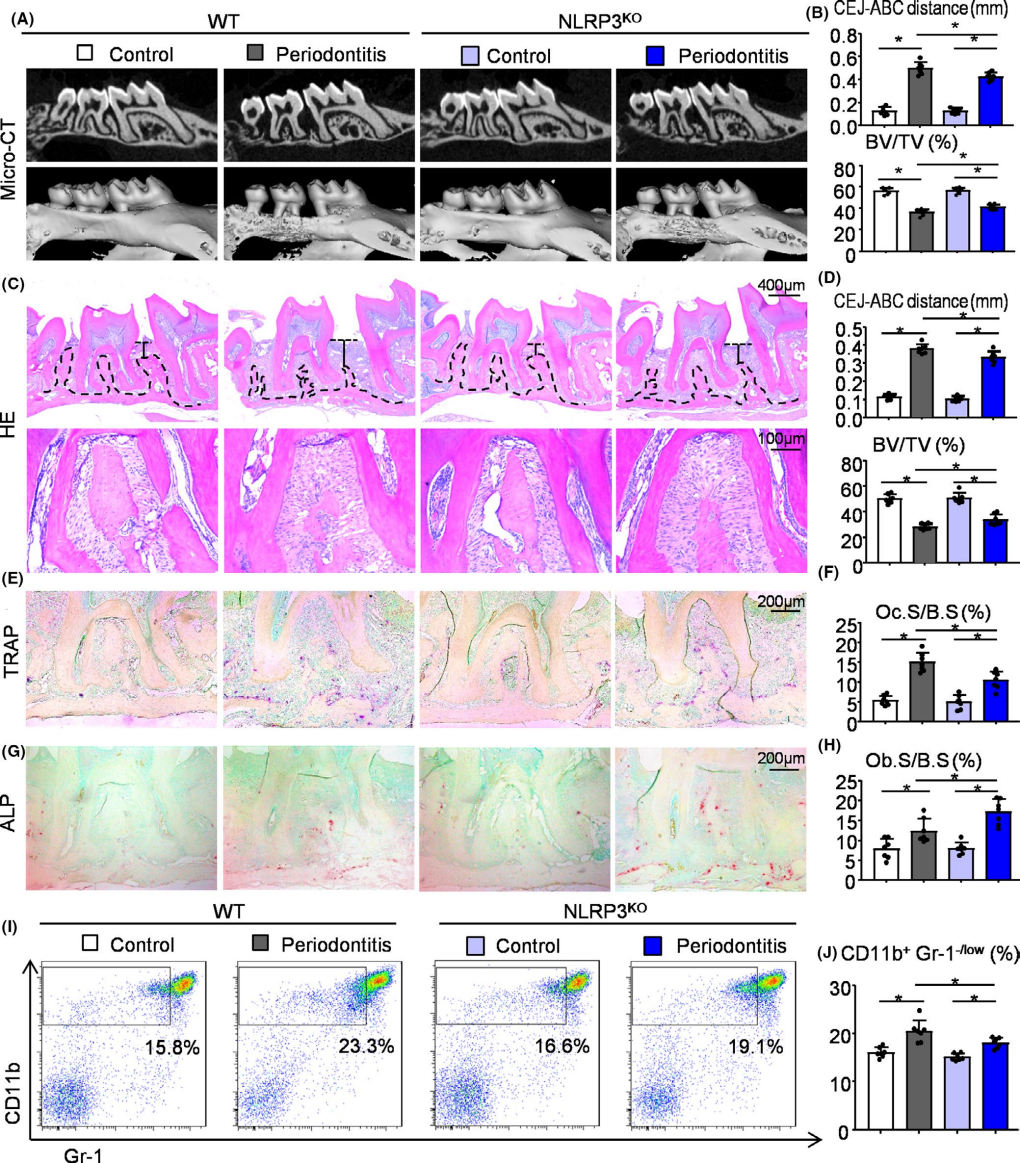
NLRP3 deficiency protects against alveolar bone loss in ligature‐induced periodontitis. Two‐month‐old NLRP3^KO^ mice and their WT littermates were used. 5‐0 silk ligature was tied around the right maxillary second molar and the other side was left untied to serve as the control group. All the mice were sacrificed 10 d after placement of the ligature. (A) Representative three‐dimensional (3D) micro‐CT scanning images (upper panels) and reconstructed sections (lower panels) along the longitudinal direction of the maxillae. (B) The distance of the cemento‐enamel junction (CEJ) to the alveolar bone crest (ABC) in mm and the percentage of BV/TV were analysed. BV/TV, which represents bone volume fraction, stands for Bone Volume over Tissue Volume. (C) Representative images of H&E‐stained paraffin sections. (D) The distance from the CEJ to ABC in mm and BV/TV (%) were determined. (E) Representative images of TRAP‐stained paraffin sections. (F) The percentage of alveolar bone surface covered by TRAP‐positive osteoclasts (Oc.S/B.S) was determined by histomorphological analysis. TRAP‐positive osteoclast surface (Oc.S/B.S) refers to the percentage of alveolar bone surface covered by mono‐ and multinucleated osteoclasts (ideally identified as TRAP‐positive). (G) Representative images of ALP‐stained paraffin sections. (H) The percentage of alveolar bone surface covered by osteoblasts (Ob.S/B.S) was determined. (I) Cells from periodontal tissues were collected and analysed by flow cytometry. Representative dot‐plot shows the population of CD11b^+^Gr‐1^−/low^ cells. (J) The percentage of CD11b^+^Gr‐1^−/low^ cells in the periodontal tissues was determined. All error bars represent SD, N = 7. One‐way ANOVA followed by Dunnett's post‐hoc multiple comparisons was performed. *P < .05 in the indicated groups

NLRP3 inflammasome has been reported to be associated with periodontitis closely.^20^, ^21^, ^22^ To explore the role of NLRP3 in periodontitis, we used a classic periodontitis model, ligature‐induced periodontitis. Two‐month‐old NLRP3^KO^ mice and their WT littermates were used and 10 days after placement of the ligature, the mice were sacrificed. μCT and histomorphometric analyses were performed to observe alveolar bone loss in NLRP3^KO^ mice and WT mice. Three‐dimensional reconstruction images and volumetric measurements from μCT analysis revealed the distance from the cemento‐enamel junction (CEJ) to the alveolar bone crest (ABC) was increased, whereas the bone volume (BV/TV) were decreased in ligature‐induced periodontitis compared with the control group (Figure 1A,B). Importantly, the distance from the CEJ to the ABC was decreased, whereas BV/TV (%) was increased, in ligature‐induced periodontitis from NLRP3^KO^ mice compared with that from WT mice (Figure 1A,B). In addition, histomorphometric measurements of H&E‐stained tissue sections, including the distance from the CEJ to the ABC and BV/TV (%), indicated the similar results as μCT in ligature‐induced periodontitis from NLRP3^KO^ mice and their WT littermates (Figure 1C,D).

Furthermore, TRAP staining was performed to evaluate osteoclast bone resorption. TRAP‐positive osteoclast surface was reduced in ligature‐induced periodontitis from NLRP3^KO^ mice than that from WT mice (Figure 1E,F). ALP staining was next performed to evaluate osteoblast bone formation. On the contrary, ALP‐positive osteoblast surface was increased in ligature‐induced periodontitis in NLRP3^KO^ mice than that in WT mice (Figure 1G,H). Both TRAP‐positive osteoclast surface and ALP‐positive osteoblast surface in ligature‐induced periodontitis were clearly increased than the control group (Figure 1E‐H). At last, flow cytometry confirmed that the percentage of CD11b^+^Gr‐1^−/low^ osteoclast precursors was lower in ligature‐induced periodontitis from NLRP3^KO^ mice than that from WT mice (Figure 1I,J).

### 3.2 NLRP3 deficiency prevents IL‐1β release and inhibits osteoclast differentiation

**FIGURE 2:**
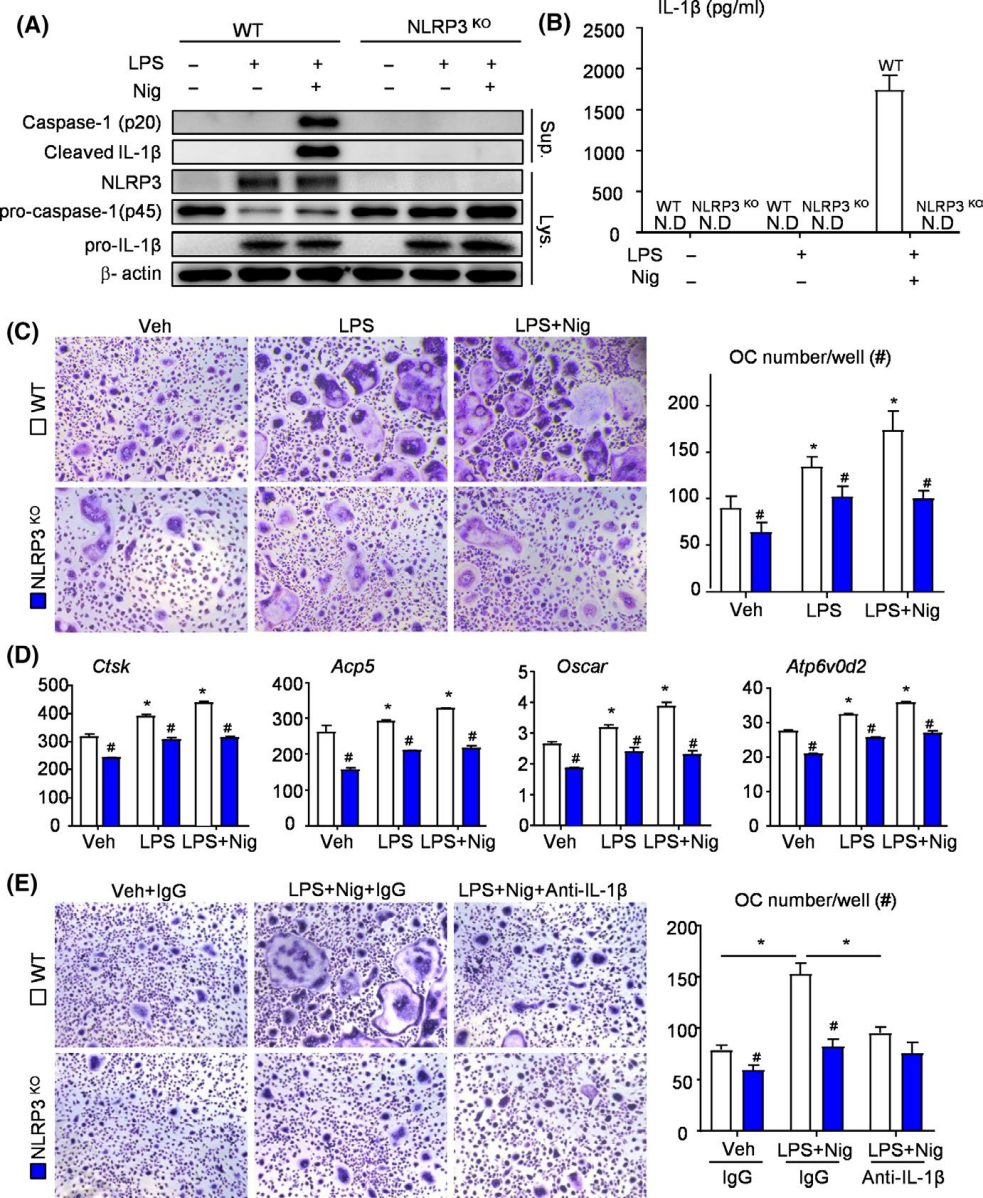
NLRP3 deficiency prevents IL‐1β release and inhibits osteoclast differentiation. (A‐D) OCPs from NLRP3^KO^ and WT mice were cultured and stimulated with or without LPS ± Nigericin during culture, as indicated in the figures. (A) Western blot of cell lysates and supernatants from OCP cultures. (B) Production of IL‐1β from OCPs stimulated with or without LPS ± Nigericin was measured by ELISA. (C) OCPs were cultured with M‐CSF and RANKL and stained for TRAP activity. TRAP^+^ OC number per well was assessed. (D) The expression levels of OC‐related genes were determined by qPCR. All the results are representative of at least three independent experiments. (E) OCPs from NLRP3^KO^ and WT mice were cultured and stimulated with or without LPS + Nigericin ±Anti‐IL‐1β during culture. OCPs were cultured with M‐CSF and RANKL and stained for TRAP activity. TRAP^+^ OC number per well was assessed. Values are mean ± SD Two‐tailed unpaired Student's *t*‐test or One‐way ANOVA followed by Dunnett's post‐hoc multiple comparisons was performed. *P < .05 vs Veh or in the indicated groups, #*P* < .05 vs WT treated with the same reagent

OCPs from NLRP3^KO^ and WT mice were used to determine the influences of deletion of NLRP3 gene on inflammatory response. After treatment with LPS and Nigericin, activated caspase‐1 p20 (an auto‐processed fragment of caspase‐1) and cleaved IL‐1β were increased in OCPs from WT mice (Figure 2A). Similarly, ELISA data showed the release of IL‐1β in OCPs from WT mice (Figure 2B). However, neither activated caspase‐1 nor cleaved IL‐1β was observed in OCPs from NLRP3^KO^ mice under the same conditions (Figure 2A,B).

To determine if NLRP3 inflammasome directly affects osteoclast differentiation, we cultured OCPs from both NLRP3^KO^ and WT mice with M‐CSF and RANKL. To activate NLRP3 inflammasome, cells were exposed to LPS and Nigericin during osteoclast formation. We found that OCPs from NLRP3^KO^ mice formed less osteoclasts than OCPs from WT mice, with or without stimulus (Figure 2C). Additionally, the expression levels of osteoclast‐related genes, including *Ctsk*, *Acp5*, *Oscar* and *Atp6v0d2*, were all significantly decreased in OCPs from NLRP3^KO^ mice compared with those from WT mice (Figure 2D). Interestingly, LPS‐primed OCPs from WT mice formed more osteoclasts and expressed higher levels of osteoclast‐related genes in the presence of Nigericin, but this effect was not observed in OCPs from NLRP3^KO^ mice (Figure 2C,D).

Given the markedly increased level of cleaved/released IL‐1β in NLRP3 activated OCPs (Figure 2A,B), which can promote osteoclast differentiation,^23^, ^24^ we next examined for changes of osteoclast formation in the presence of IL‐1β neutralizing antibody. As shown in Figure 2E, OCPs from WT mice receiving IL‐1β neutralizing antibody plus LPS and Nigericin formed less osteoclasts than LPS and Nigericin‐ treated OCPs. However,similar results did not appear in OCPs from NLRP3^KO^ mice (Figure 2E). Therefore, NLRP3 may mediated osteoclast differentiation through IL‐1β production.

### 3.3 MCC950 decreases osteoclast differentiation through NLRP3 inflammasome inhibition

**FIGURE 3:**
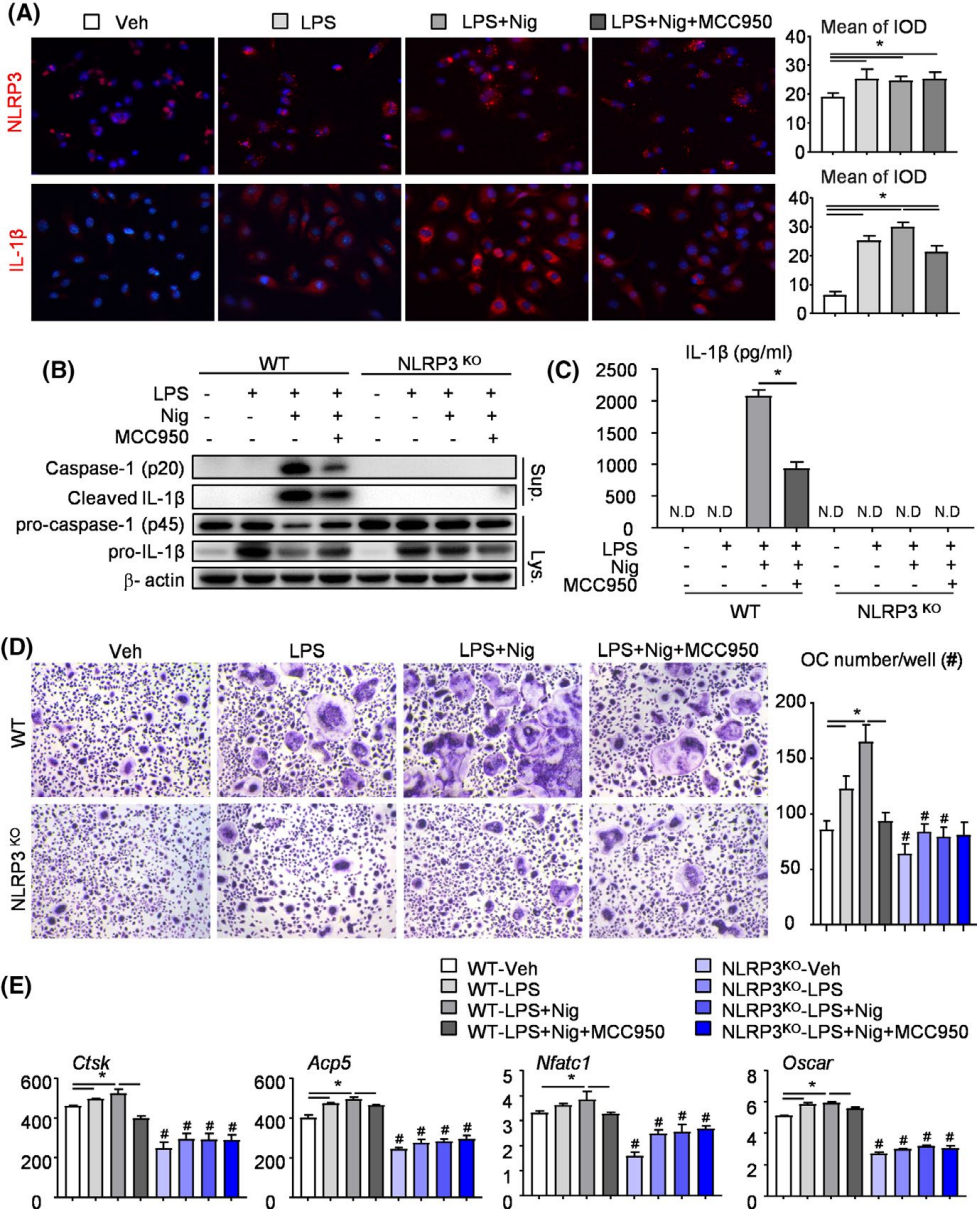
MCC950 decreases osteoclast differentiation through NLRP3 inflammasome inhibition. (A) OCPs from WT mice were cultured and stimulated with or without LPS ± MCC950 ± Nigericin during culture. OCPs were then subjected to IF staining with anti‐NLRP3 and anti‐IL‐1β Abs. Representative images and analyses of integrated optical density (IOD) for NLRP3 and IL‐1β were showed. (B‐E) OCPs from NLRP3^KO^ mice and their WT littermates were cultured and stimulated with or without LPS ± MCC950 ± Nigericin during culture, as indicated in the figures. (B) Western blot of cell lysates and supernatants from OCP cultures. (C) Production of IL‐1β from OCPs was measured by ELISA. (D) OCPs were cultured with M‐CSF and RANKL and treated with or without LPS ± Nigericin ± MCC950 and finally stained for TRAP activity. TRAP^+^ OC number was assessed. (E) The expression levels of OC‐related genes were determined by qPCR. All the results are representative of at least three independent experiments. Values are mean ± SD One‐way ANOVA followed by Dunnett's post‐hoc multiple comparisons was performed. *P < .05 vs Veh or in the indicated groups, #*P* < .05 vs WT treated with the same reagent

MCC950, as a potent, selective, small molecule inhibitor of NLRP3, can inhibit canonical NLRP3 activation.^25^ Considering the decreased osteoclast differentiation in NLRP3^KO^ mice (Figures 1E and 2C‐E), we speculate that MCC950 may inhibit osteoclast differentiation through NLRP3 inhibition. The effects of MCC950 on NLRP3 inflammasome activation were detected in OCPs from WT mice, which were first primed with LPS, then pre‐treated with MCC950 and lastly stimulated with Nigericin. IF staining revealed that the protein levels of NLRP3 and IL‐1β were increased in LPS‐ or LPS plus Nigericin‐ induced OCPs compared with vehicle‐treated OCPs (Figure 3A). Importantly, MCC950 partially blocked the increased level of IL‐1β in LPS plus Nigericin‐ treated OCPs (Figure 3A).

To further determine whether MCC950 targets are NLRP3 or other molecules, the effects of MCC950 on NLRP3 inflammasome activation were detected in OCPs from NLRP3^KO^ mice and their WT littermates. The protein levels of caspase‐1 p20, cleaved IL‐1β and released IL‐1β were evidently reduced in supernatants from MCC950‐treated WT OCPs (Figure 3B,C). By contrast, the protein levels of caspase‐1 p20, cleaved IL‐1β and released IL‐1β were undetectable in supernatants from MCC950‐treated NLRP3^KO^ OCPs (Figure 3B,C). In order to observe the effect of MCC950 on osteoclast differentiation intuitively, OCPs were cultured with M‐CSF and RANKL. TRAP staining showed that both LPS‐ primed and Nigericin‐ stimulated promoted osteoclast differentiation in OCPs from WT mice, but MCC950 played the opposite role (Figure 3D). Similar trends appeared in real‐time RT‐PCR, which was used to examine the expression levels of osteoclast‐related genes including *Ctsk*, *Acp5*, *Nfatc1* and *Oscar* (Figure 3E). Importantly, MCC950 could not exert the similar effects on OCPs from NLRP3^KO^ mice (Figure 3D,E), suggesting MCC950 may decrease osteoclast differentiation through NLRP3 inflammasome inhibition.

### 3.4 MCC950 reduces the number of Lysm‐Cre^+^ osteoclast precursors in ligature‐induced periodontitis

**FIGURE 4:**
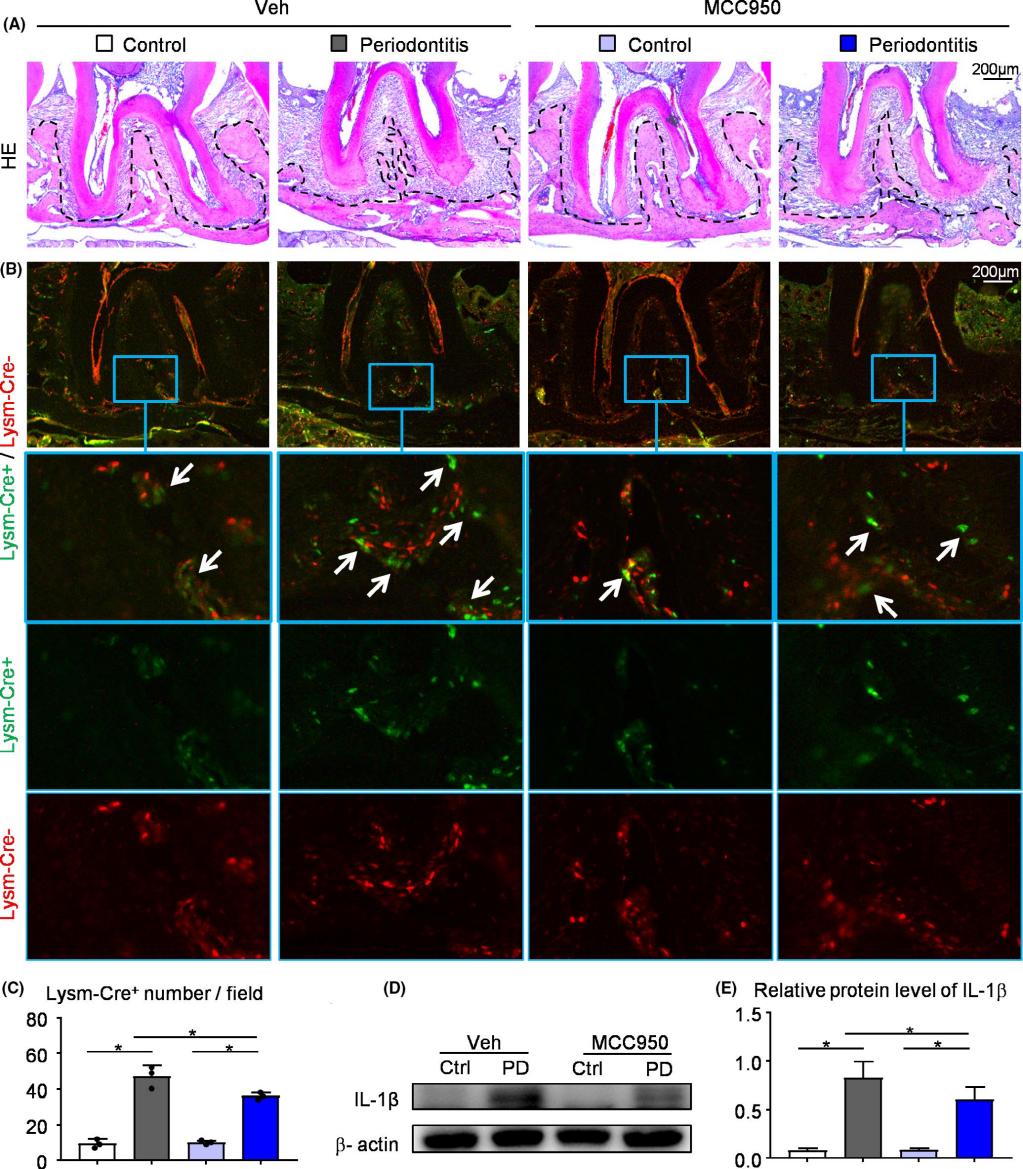
MCC950 reduces the number of Lysm‐Cre^+^ osteoclast precursors in ligature‐induced periodontitis. (A‐C) Frozen sections of maxillary second molar from 2‐month‐old Lysm‐cre/Rosa^nTnG^ mice received i.p. injection of MCC950 or PBS were used, N = 4‐5. Representative images of H&E‐stained frozen sections of maxillae (A) and adjacent unstained sections under fluorescence microscopy (B). Images show Lysm‐Cre^+^ cells (GFP, green) and Cre^–^ cells (tdTomato, red) in maxillae. The blue boxes are shown enlarged in the lower panels. White arrows indicate the Lysm‐Cre^+^ cells. (C) Lysm‐Cre^+^ number per field was assessed. (D) Protein from periodontal tissues was Western blotted to determine the expression of IL‐1β, with β‐actin as the loading control. (E) IL‐1β protein level relative to that of β‐actin was assessed by densitometric analysis. All the results are representative of at least three independent experiments. Values are mean ± SD One‐way ANOVA followed by Dunnett's post‐hoc multiple comparisons was performed. *P < .05 in the indicated groups

We next used Lysm‐Cre/Rosa^nTnG^ mouse, a double‐fluorescent Cre reporter mouse, to observe the effects of MCC950 on Lysm‐Cre^+^ osteoclast precursors in vivo. In Lysm‐Cre/Rosa^nTnG^ mouse, all Lysm‐Cre^+^ cells and their descendants express GFP, whereas all Lysm‐Cre^−^ cells express tdTomato.^26^ We examined the number of Lysm‐Cre^+^ cells in ligature‐induced periodontitis and control group from Lysm‐cre/Rosa^nTnG^ mice receiving MCC950 or vehicle by frozen embedded sections of maxilla. It was clear that more Lysm‐Cre^+^ cells were found in ligature‐induced periodontitis than control group (Figure 4A‐C). Notably, MCC950‐treated periodontitis group reduced the number of Lysm‐Cre^+^ cells compared with vehicle‐treated periodontitis group (Figure 4A‐C).

Proteins from periodontal tissues around maxillary second molar were extracted and examined for the expression level of IL‐1β, which represents the activation state of NLRP3 inflammasome. We found that the expression level of IL‐1β was increased in periodontitis group compared with that in control group. Importantly, the expression level of IL‐1β was decreased in MCC950‐treated group compared with that in vehicle‐treated group, as expected (Figure 4D,E).

### 3.5 MCC950 inhibits Lysm‐Cre^+^ osteoclast precursors differentiation into osteoclast

**FIGURE 5:**
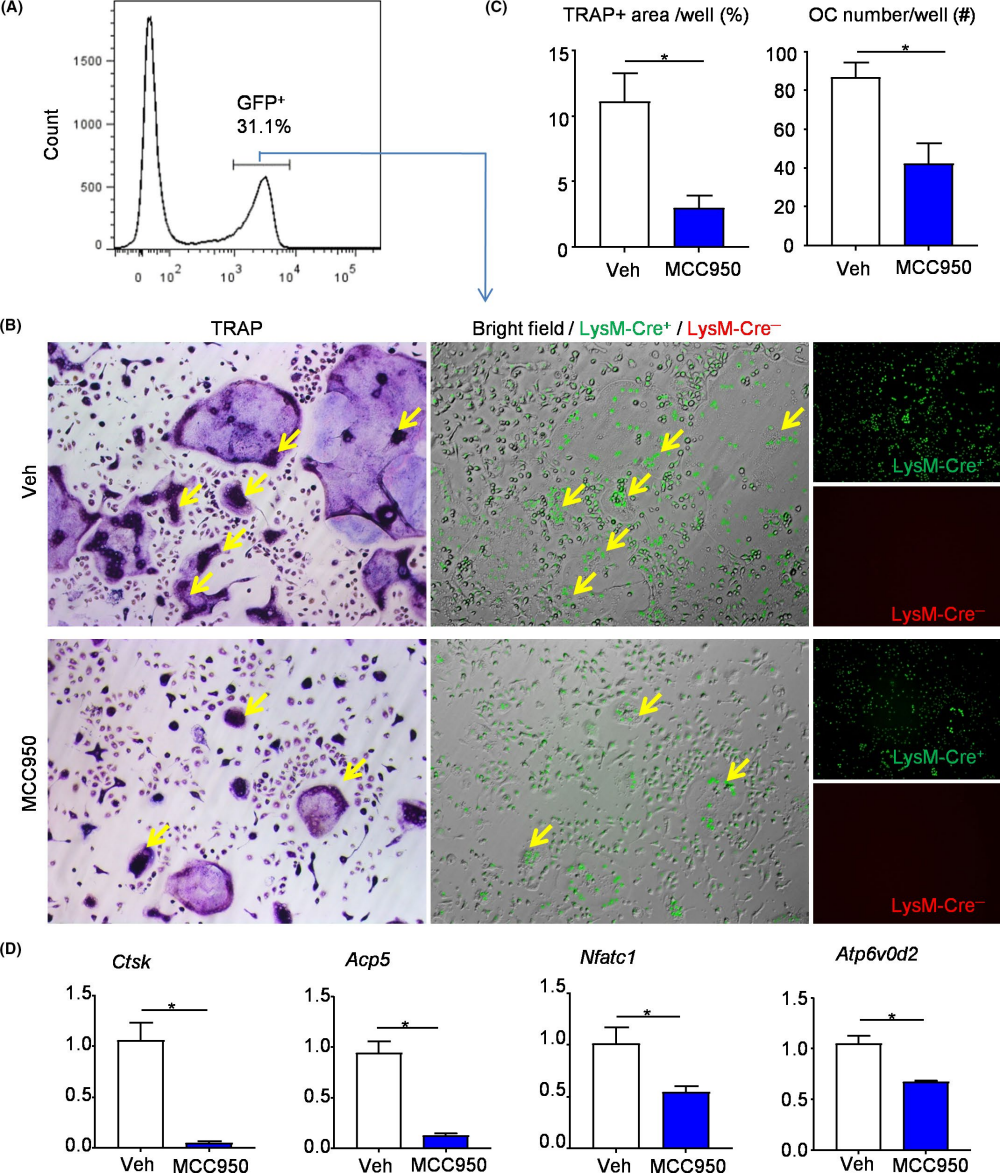
MCC950 inhibits Lysm‐Cre^+^ osteoclast precursors differentiation into osteoclast. Lysm‐Cre^+^ cells (GFP, green) from bone marrow were sorted out by flow cytometry after injection with MCC950 or vehicle. (A) Flow diagram of distinguishing Lysm‐Cre^+^ cells (GFP^+^). (B) The selected Lysm‐Cre^+^ cells were cultured with M‐CSF and RANKL and stained for TRAP activity. Same field of TRAP‐stained images (left panel), fluorescence images (right panel) and merged images of bright field and fluorescence images (middle panel) were shown. Yellow arrows show TRAP‐positive and Lysm‐Cre^+^ cells. (C) TRAP^+^ OC area and TRAP^+^ OC number were assessed. (D) The expression levels of OC‐related genes were determined by qPCR. All the results are representative of at least three independent experiments. Values are mean ± SD Two‐tailed unpaired Student's *t*‐test was performed. *P < .05 in the indicated groups

To further determine the effects of MCC950 on osteoclast differentiation, Lysm‐Cre‐GFP^+^ cells from bone marrow were sorted out by flow cytometry after treated with MCC950 or vehicle (Figure 5A). First, Lysm‐Cre‐GFP^+^ cells were subjected to osteoclastogenic assay with M‐CSF and RANKL. TRAP staining was performed to observe osteoclast formation (Figure 5B). Both TRAP^+^ osteoclast area and TRAP^+^ osteoclast number were decreased clearly from the mice receiving MCC950 compared with that receiving vehicle (Figure 5B,C). Besides, Lysm‐Cre‐GFP^+^ cells were also used to test the expression levels of osteoclast‐related genes by real‐time RT‐PCR. The expression levels of genes, including *Ctsk*, *Acp5*, *Nfatc1* and *Atp6v0d2,* were appreciably downregulated from the mice receiving MCC950 compared with that receiving vehicle (Figure 5D).

### 3.6 MCC950 protects against alveolar bone loss in ligature‐induced periodontitis by inhibiting the NLRP3 inflammasome activation and osteoclast differentiation

**FIGURE 6:**
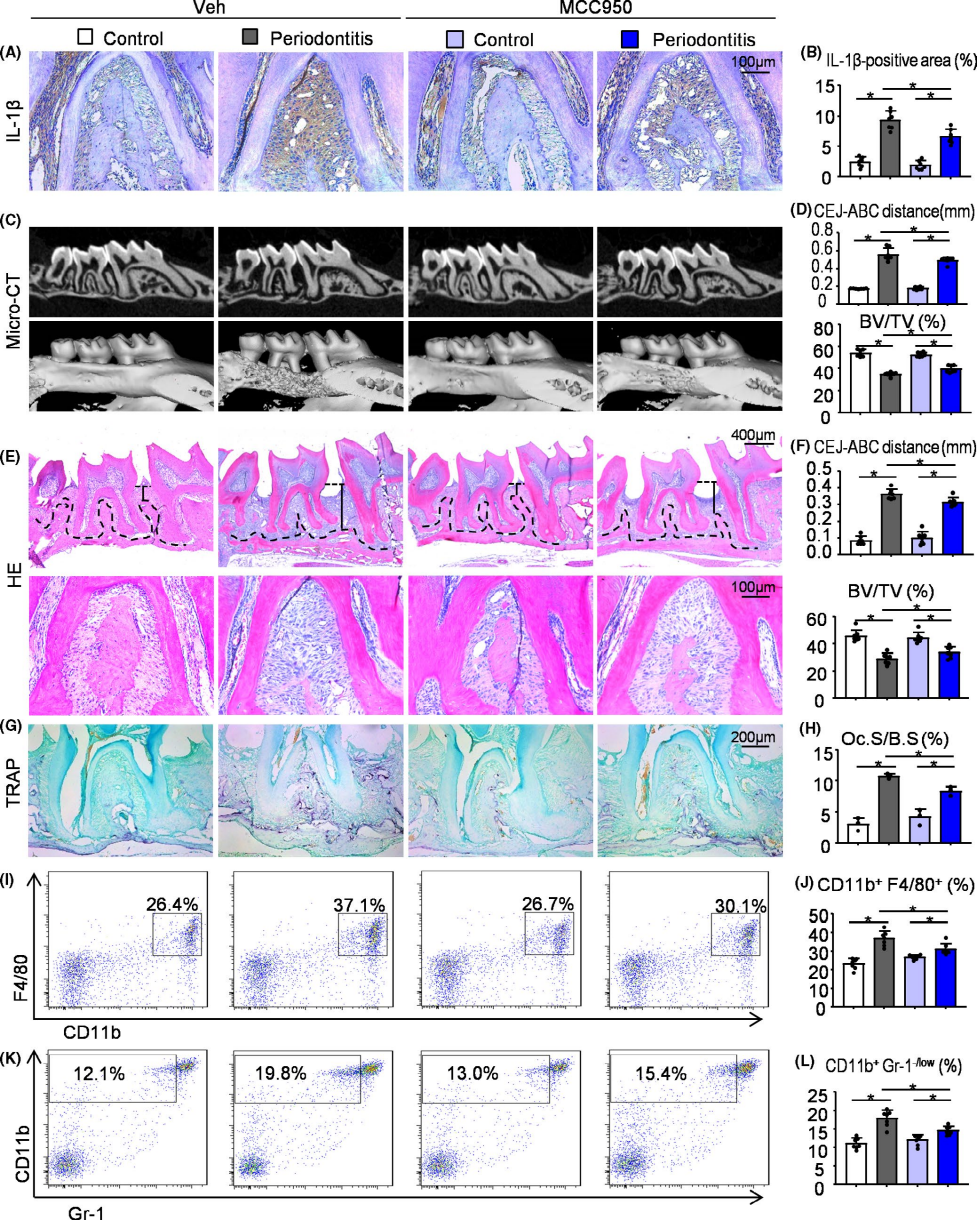
MCC950 protects against alveolar bone loss in ligature‐induced periodontitis by inhibiting the NLRP3 inflammasome activation and osteoclast differentiation. Two‐month‐old WT mice received i.p. injection of MCC950 or vehicle were used. 5‐0 silk ligature was tied around the right maxillary second molar and the other side was left untied to serve as the control group. All the mice were sacrificed 10 d after placement of the ligature. (A) Representative IHC images for IL‐1β in the root furcation of maxillary second molar. (B) The percentage of IL‐1β‐positive area was calculated. (C) Representative three‐dimensional (3D) micro‐CT scanning images (upper panels) and reconstructed sections (lower panels) along the longitudinal direction of the maxillae. (D) The distance of the cemento‐enamel junction (CEJ) to the alveolar bone crest (ABC) in mm and BV/TV (%) were analysed. (E) Representative images of H&E‐stained paraffin sections. (F) The distance from the CEJ to ABC in mm and BV/TV (%) were determined. (G) Representative images of TRAP‐stained paraffin sections. (H) The percentage of alveolar bone surface covered by TRAP‐positive osteoclasts (Oc.S/B.S) was determined by histomorphological analysis. Cells from periodontal tissues were collected and analysed by flow cytometry. Representative dot‐plot shows the population of CD11b^+^F4/80^+^ cells (I) and CD11b^+^ Gr‐1^−/low^ cells (K). The percentage of CD11b^+^F4/80^+^ cells (J) and CD11b^+^Gr‐1^−/low^ cells (L) was measured. All error bars represent SD, N = 7. One‐way ANOVA followed by Dunnett's post‐hoc multiple comparisons was performed. *P < .05 in the indicated groups

To demonstrate if MCC950 protects against alveolar bone loss in ligature‐induced periodontitis, 2‐month‐old WT mice were received i.p. injection of MCC950 or vehicle, and then subjected to ligature‐induced periodontitis group or control group. The positive area of IL‐1β in the second molar root furcation was decreased in ligature‐induced periodontitis mice receiving MCC950 compared with that receiving vehicle (Figure 6A,B). It is suggested that MCC950 could inhibit the activation of NLRP3 inflammasomes in ligature‐induced periodontitis. Importantly, BV/TV (%) was increased, whereas the distance from the CEJ to ABC was decreased in ligature‐induced periodontitis mice receiving MCC950 compared with that receiving vehicle (Figure 6C‐F). TRAP‐positive osteoclast surface was reduced in ligature‐induced periodontitis mice receiving MCC950 compared with that receiving vehicle (Figure 6G,H). Meanwhile, we used flow cytometry to examine cell populations of periodontal tissues around maxillary second molar from ligature‐induced periodontitis receiving MCC950 or vehicle. The results revealed that the percentage of CD11b^+^F480^+^ macrophages (Figure 6I,J) and CD11b^+^Gr‐1^−/low^ osteoclast precursors (Figure 6K,L) were both lower in ligature‐induced periodontitis mice receiving MCC950 compared with that receiving vehicle.

## 4 DISCUSSION

**FIGURE 7:**
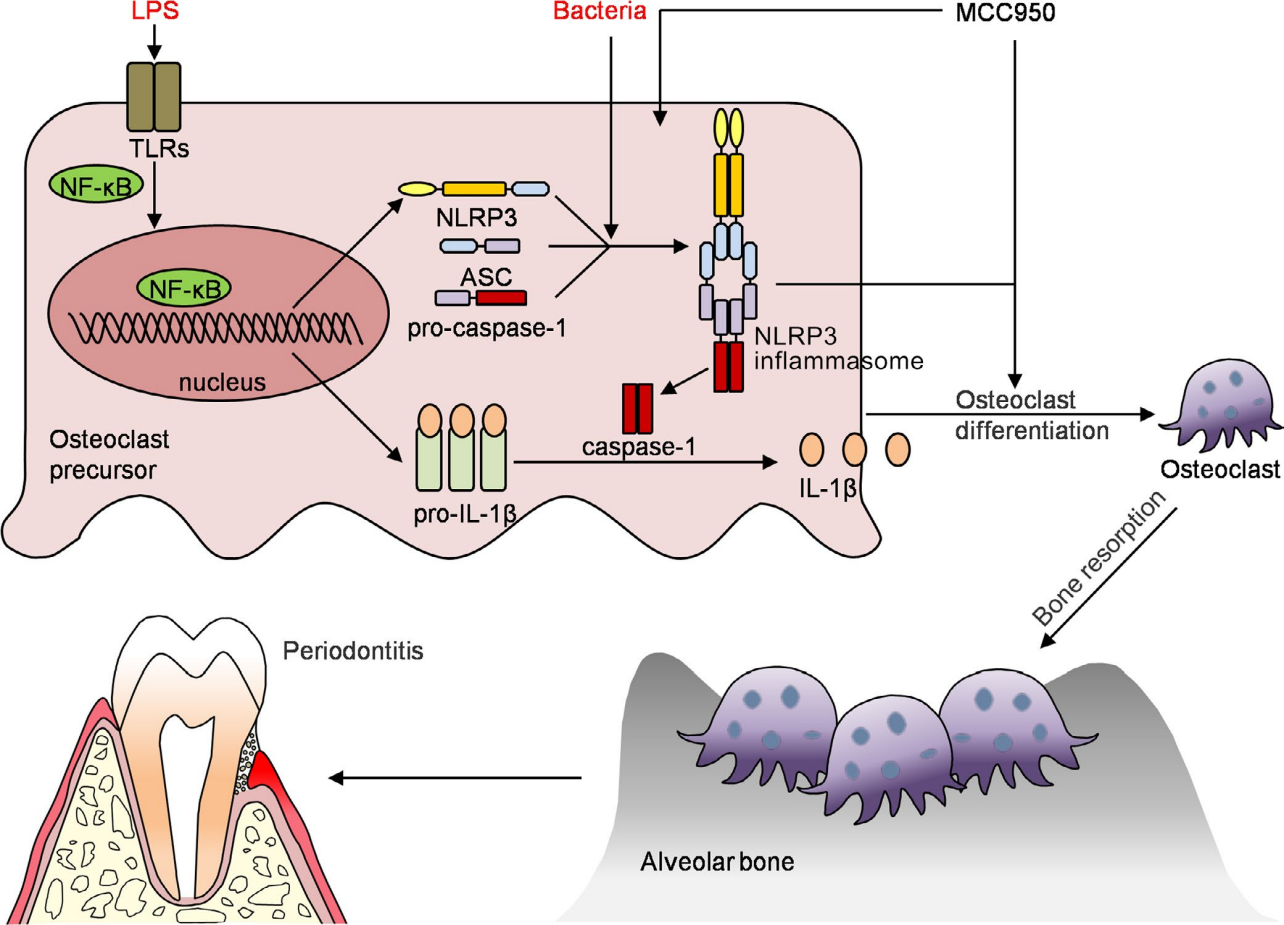
A model depicting how NLRP3 regulating alveolar bone loss in ligature‐induced periodontitis. Bacterial infection in periodontal tissues leads to the activation of NLRP3 inflammasome in osteoclast precursor cells, which promotes osteoclast differentiation and alveolar bone resorption. Thus, NLRP3 inflammasome contribute significantly to the pathologic bone loss in periodontitis. MCC950, a specific inhibitor of the NLRP3 inflammasome, inhibits osteoclast differentiation, thereby reduces alveolar bone loss in periodontitis

The main findings of the present study are as follows (Figure 7): (i) NLRP3 regulates alveolar bone loss in ligature‐induced periodontitis by promoting osteoclastic differentiation; (ii) MCC950 suppresses alveolar bone loss with reduced IL‐1β activation and osteoclast differentiation in ligature‐induced periodontitis.

Several studies have reported the important role of NLRP3 in periodontitis, with the focus on the function of NLRP3 on macrophage^12^ or periodontal ligament cells.^27^ To the best of our knowledge, the first report focused on the effects of NLRP3 on osteoclast differentiation in periodontitis, found that NLRP3 inflammasome does not have a relevant role in the inflammatory bone resorption.^28^ These results are in contrast with those of a study that used a Porphyromonas gingivalis oral colonization model of experimental periodontitis, which showed a significant attenuation of bone resorption in NLRP3‐deficient mice.^12^ Thereafter, another well‐done and extensive study used ageing‐related periodontitis as an in vivo model, suggested that NLRP3 plays an important role in osteoclastogenesis during ageing.^29^ However, ageing is a very complex process. Besides NLRP3 mediated inflammation, osteoclast differentiation in ageing has been affected by many other factors. Therefore, in our study, we used ligature‐induced periodontitis models of NLRP3^KO^ and their wildtype littermates to compare their alveolar bone phenotypes with the focus on osteoclast differentiation. We found that the number of osteoclast precursors, osteoclast differentiation and alveolar bone loss were reduced in NLRP3^KO^ mice compared with WT littermates. Importantly, we used the Lysm‐Cre/Rosa^nTnG^ mice treated with or without MCC950, which could offer a better tool to study the relation of NLRP3 and osteoclast differentiation in vitro and in vivo.

MCC950, as an inhibitor of NLRP3, has been widely verified for the treatment of many inflammatory diseases, such as inflammatory bowel diseases,^30^ Parkinson's disease^31^ and Autoimmune encephalomyelitis,^32^ due to its low dose and minor side effects. MCC950 has been used to develop improved drug candidate which are planned to carry out human clinical trials for Parkinson's disease in recent years. In addition, several studies have proved that MCC950 has therapeutic effects on oral diseases. MCC950 inhibited NLRP3 activation and M1 macrophage polarization in human periodontal ligament cells (hPDLCs) from inflammatory root resorption.^13^ MCC950 prevented inflammatory response in THP‐1 macrophage‐like cells, which was caused by periodontopathic bacteria.^20^ Moreover, MCC950 improved anti‐tumour immune responses in head and neck squamous cell carcinoma.^33^ Most of these studies focus on the cellular level and lack in vivo studies. In this study, we used ligature‐induced periodontitis murine model to demonstrate that MCC950 may play a positive role in treating periodontitis. Furthermore, Lysm‐Cre/Rosa^nTnG^ mice were used to establish ligature‐induced periodontitis and demonstrate that MCC950 acts directly on osteoclast precursors, and thereby prevents osteoclast differentiation and alveolar bone loss in periodontitis.

Qu et al^34^ reported that expression of *Nlrp3^D301N^* in myeloid cells or osteoclasts causes constitutive activation of the NLRP3 inflammasome, which is sufficient to cause higher number of OCPs, more OC differentiation and bone resorption, by using *Nlrp3^D301N/+;LysM^* mice and *Nlrp3^D301N/+;CatK^* mice. Although NLRP3 is mainly expressed in monocytes/macrophages,^35^ it is also found in mesenchymal stem cells ^36^ and osteoblasts.^37^ Up‐regulation of NLRP3 in mesenchymal stem cells inhibits osteogenic differentiation.^38^ In osteoblasts, MCC950 inhibits NLRP3 inflammasome‐mediated pyroptosis and promotes early osteogenic differentiation.^39^ Correspondingly, our data also found that NLRP3^KO^ mice had increased osteoblast activity in ligature‐induced periodontitis, compared with WT mice. Thus, it is possible that the increased alveolar bone volume caused by NLRP3 deficiency may be partly due to the effects of NLRP3 on osteoblast differentiation. In the future, we may generate myeloid specific NLRP3‐overexpression or knockout mice by crossing NLRP3‐Tg mice or NLRP3‐floxed mice with Lysm‐cre or CX3CR1‐cre mice and then establishing ligature‐induced periodontitis in the absence and presence of the NLRP3 inhibitor, MCC950. These studies will definitively show a role of NLRP3 inflammasome on osteoclast differentiation in periodontitis.

In summary, by using ligature‐induced periodontitis murine models, we have demonstrated that both NLRP3 deficiency and MCC950 reduce the number of osteoclast precursors and prevent osteoclast differentiation, thereby protect against alveolar bone loss in ligature‐induced periodontitis. Thus, our results suggest that NLRP3 regulates bone resorption in periodontitis by mediating osteoclast differentiation. MCC950 may be used as a potential drug to treat periodontitis in the future.

## CONFLICT OF INTEREST

The authors declare no competing financial interests.

## AUTHOR CONTRIBUTIONS

Authors’ roles: Study design: YC, HW, WS and HY. Study conduct: YC, QY, CL, YC, WZ, WL, HC, HW, WS and HY. Data collection: YC, QY, CL, YC, WL and HC. Data analysis: YC, WS and HY. Data interpretation: YC, HW, WS and HY. Drafting manuscript: YC, WS and HY. Revising manuscript content: YC, WS and HY. Approving final version of manuscript: YC, QY, CL, YC, WZ, WL, HC, HW, WS and HY. WS and HY take responsibility for the integrity of the data analysis.

## ORIGINAL PUBLICATION

The research is original, not under publication consideration elsewhere.

## Supporting information

Table S1

## Data Availability

The authors declare that all data supporting the findings of this study are available within the article or are available from the corresponding author upon request.

## References

[1] Nascimento P , Castro M , Magno M , et al. Association between periodontitis and cognitive impairment in adults: a systematic review. Front Neurol. 2019;10:323. 31105630 10.3389/fneur.2019.00323 PMC6492457

[2] Trofin E , Monsarrat P , Kémoun P . Cell therapy of periodontium: From animal to human? Front Physiol. 2013;4:325. 24298258 10.3389/fphys.2013.00325 PMC3828527

[3] Hoare A , Soto C , Rojas‐Celis V , Bravo D . Chronic inflammation as a link between periodontitis and carcinogenesis. Mediators Inflamm. 2019;2019:1029857. 31049022 10.1155/2019/1029857 PMC6458883

[4] Kelley N , Jeltema D , Duan Y , He Y . The NLRP3 inflammasome: an overview of mechanisms of activation and regulation. Int J Mol Sci. 2019;20:3328. 10.3390/ijms20133328 PMC6651423 31284572

[5] Kim SY , Heo S , Kim SH , et al. Suppressive effects of dehydrocostus lactone on the toll‐like receptor signaling pathways. Int Immunopharmacol. 2020;78:106075. 31812722 10.1016/j.intimp.2019.106075

[6] Liu D , Zeng X , Li X , et al. Advances in the molecular mechanisms of NLRP3 inflammasome activators and inactivators. Biochem Pharmacol. 2020;175:113863. 32081791 10.1016/j.bcp.2020.113863

[7] Wang L , Hauenstein AV . The NLRP3 inflammasome: mechanism of action, role in disease and therapies. Mol Aspects Med. 2020;76:100889. 32859386 10.1016/j.mam.2020.100889

[8] Ma M , Pei Y , Wang X , Feng J , Zhang Y , Gao MQ . LncRNA XIST mediates bovine mammary epithelial cell inflammatory response via NF‐κB/NLRP3 inflammasome pathway. Cell Prolif. 2019;52(1):e12525. 30362186 10.1111/cpr.12525 PMC6430464

[9] Shao BZ , Xu ZQ , Han BZ , Su DF , Liu C . NLRP3 inflammasome and its inhibitors: a review. Front Pharmacol. 2015;6:262. 26594174 10.3389/fphar.2015.00262 PMC4633676

[10] Jiang H , He H , Chen Y , et al. Identification of a selective and direct NLRP3 inhibitor to treat inflammatory disorders. J Exp Med. 2017;214(11):3219‐3238. 29021150 10.1084/jem.20171419 PMC5679172

[11] Pan H , Lin Y , Dou J , et al. Wedelolactone facilitates Ser/Thr phosphorylation of NLRP3 dependent on PKA signalling to block inflammasome activation and pyroptosis. Cell Prolif. 2020;53(9):e12868. 32656909 10.1111/cpr.12868 PMC7507381

[12] Yamaguchi Y , Kurita‐Ochiai T , Kobayashi R , Suzuki T , Ando T . Regulation of the NLRP3 inflammasome in Porphyromonas gingivalis‐accelerated periodontal disease. Inflamm Res. 2017;66(1):59‐65. 27665233 10.1007/s00011-016-0992-4

[13] Zhang J , Liu X , Wan C , et al. NLRP3 inflammasome mediates M1 macrophage polarization and IL‐1β production in inflammatory root resorption. J Clin Periodontol. 2020;47(4):451‐460. 31976565 10.1111/jcpe.13258

[14] Wu X , Lu M , Ding S , Zhong Q . Tripartite motif 31 alleviates IL‐1ß secretion via promoting the ubiquitination of pyrin domain domains‐containing protein 3 in human periodontal ligament fibroblasts. Odontology. 2020;108(3):424‐432. 32323100 10.1007/s10266-020-00519-7

[15] Guarda G , Zenger M , Yazdi AS , et al. Differential expression of NLRP3 among hematopoietic cells. J Immunol. 2011;186(4):2529‐2534. 21257968 10.4049/jimmunol.1002720

[16] Rao Z , Chen X , Wu J , et al. Vitamin D receptor inhibits NLRP3 activation by impeding its BRCC3‐mediated deubiquitination. Front Immunol. 2019;10:2783. 31866999 10.3389/fimmu.2019.02783 PMC6904361

[17] Sun W , Wu J , Huang L , et al. PTHrP nuclear localization and carboxyl terminus sequences modulate dental and mandibular development in part via the action of p27. Endocrinology. 2016;157(4):1372‐1384. 26859332 10.1210/en.2015-1555

[18] Wang H , Chen Y , Li W , et al. Effect of VEGFC on lymph flow and inflammation‐induced alveolar bone loss. J Pathol. 2020;251(3):323‐335. 32418202 10.1002/path.5456 PMC10587832

[19] Wang H , Lv C , Gu Y , et al. Overexpressed Sirt1 in MSCs promotes dentin formation in Bmi1‐deficient mice. J Dent Res. 2018;97(12):1365‐1373. 29932801 10.1177/0022034518781509

[20] Kawahara Y , Kaneko T , Yoshinaga Y , et al. Effects of sulfonylureas on periodontopathic bacteria‐induced inflammation. J Dent Res. 2020;99(7):830‐838. 32202959 10.1177/0022034520913250

[21] Cheng R , Liu W , Zhang R , Feng Y , Bhowmick NA , Hu T . Porphyromonas gingivalis‐derived lipopolysaccharide combines hypoxia to induce caspase‐1 activation in periodontitis. Front Cell Infect Microbiol. 2017;7:474. 29184853 10.3389/fcimb.2017.00474 PMC5694474

[22] Park E , Na HS , Song YR , Shin SY , Kim YM , Chung J . Activation of NLRP3 and AIM2 inflammasomes by Porphyromonas gingivalis infection. Infect Immun. 2014;82(1):112‐123. 24126516 10.1128/IAI.00862-13 PMC3911849

[23] Lee B , Kim TH , Jun JB , et al. Direct inhibition of human RANK+ osteoclast precursors identifies a homeostatic function of IL‐1beta. J Immunol. 2010;185(10):5926‐5934. 20935210 10.4049/jimmunol.1001591 PMC3016227

[24] Shiratori T , Kyumoto‐Nakamura Y , Kukita A , et al. IL‐1β induces pathologically activated osteoclasts bearing extremely high levels of resorbing activity: a possible pathological subpopulation of osteoclasts, accompanied by suppressed expression of kindlin‐3 and talin‐1. J Immunol. 2018;200(1):218‐228. 29141864 10.4049/jimmunol.1602035

[25] Coll RC , Robertson AA , Chae JJ , et al. A small‐molecule inhibitor of the NLRP3 inflammasome for the treatment of inflammatory diseases. Nat Med. 2015;21(3):248‐255. 25686105 10.1038/nm.3806 PMC4392179

[26] Dallas SL , Xie Y , Shiflett LA , Ueki Y . Mouse Cre models for the study of bone diseases. Current Osteoporos Rep. 2018;16(4):466‐477. 10.1007/s11914-018-0455-7 PMC6397767 29934753

[27] Tan Y , Chen J , Jiang Y , et al. The anti‐periodontitis action of metformin via targeting NLRP3 inflammasome. Arch Oral Biol. 2020;114:104692. 32305805 10.1016/j.archoralbio.2020.104692

[28] Rocha FRG , Delitto AE , de Souza JAC , González‐Maldonado LA , Wallet SM , Rossa JC . Relevance of caspase‐1 and Nlrp3 inflammasome on inflammatory bone resorption in a murine model of periodontitis. Sci Rep. 2020;10(1):7823. 32385413 10.1038/s41598-020-64685-y PMC7210885

[29] Zang Y , Song JH , Oh SH , et al. Targeting NLRP3 inflammasome reduces age‐related experimental alveolar bone loss. J Dent Res. 2020;99(11):1287‐1295. 32531176 10.1177/0022034520933533

[30] Perera AP , Fernando R , Shinde T , et al. MCC950, a specific small molecule inhibitor of NLRP3 inflammasome attenuates colonic inflammation in spontaneous colitis mice. Sci Rep. 2018;8(1):8618. 29872077 10.1038/s41598-018-26775-w PMC5988655

[31] Gordon R , Albornoz EA , Christie DC , et al. Inflammasome inhibition prevents α‐synuclein pathology and dopaminergic neurodegeneration in mice. Sci Transl Med. 2018;10(465):eaah4066. 30381407 10.1126/scitranslmed.aah4066 PMC6483075

[32] Hou B , Zhang Y , Liang P , et al. Inhibition of the NLRP3‐inflammasome prevents cognitive deficits in experimental autoimmune encephalomyelitis mice via the alteration of astrocyte phenotype. Cell Death Dis. 2020;11(5):377. 32415059 10.1038/s41419-020-2565-2 PMC7229224

[33] Chen L , Huang CF , Li YC , et al. Blockage of the NLRP3 inflammasome by MCC950 improves anti‐tumor immune responses in head and neck squamous cell carcinoma. Cell Mol Life Sci. 2018;75(11):2045‐2058. 29184980 10.1007/s00018-017-2720-9 PMC11105265

[34] Qu C , Bonar SL , Hickman‐Brecks CL , et al. NLRP3 mediates osteolysis through inflammation‐dependent and ‐independent mechanisms. FASEB journal. 2015;29(4):1269‐1279. 25477279 10.1096/fj.14-264804 PMC4396608

[35] Greta G . Differential expression of NLRP3 among hematopoietic cells. J Immunol. 2011;4(186):2529‐2534. 10.4049/jimmunol.1002720 21257968

[36] Ahn JS , Seo Y , Oh SJ , et al. The activation of NLRP3 inflammasome potentiates the immunomodulatory abilities of mesenchymal stem cells in a murine colitis model. BMB Rep. 2020;53(6):329‐334. 32475381 10.5483/BMBRep.2020.53.6.065 PMC7330809

[37] McCall SH , Sahraei M , Young AB , et al. Osteoblasts express NLRP3, a nucleotide‐binding domain and leucine‐rich repeat region containing receptor implicated in bacterially induced cell death. J Bone Miner Res. 2008;23(1):30‐40. 17907925 10.1359/JBMR.071002 PMC2663588

[38] Xu L , Zhang L , Wang Z , et al. Melatonin suppresses estrogen deficiency‐induced osteoporosis and promotes osteoblastogenesis by inactivating the NLRP3 inflammasome. Calcif Tissue Int. 2018;103(4):400‐410. 29804160 10.1007/s00223-018-0428-y

[39] Liu S , Du J , Li D , et al. Oxidative stress induced pyroptosis leads to osteogenic dysfunction of MG63 cells. J Mol Histol. 2020;51(3):221‐232. 32356234 10.1007/s10735-020-09874-9

