# Supplementary material for: NLRP3 regulates alveolar bone loss in ligature‐induced periodontitis by promoting osteoclastic differentiation
Source: Cell Prolif. 2020 Dec 31;54(2):e12973. doi: 10.1111/cpr.12973 (PMC7849172; doi:10.1111/cpr.12973)
Supplement: Supplementary file 1 — Table S1 [file CPR-54-e12973-s001.docx]

**Table S1.** The sequences of primers used.

| Name | F/R | Sequences |
| --- | --- | --- |
| *Gapdh* | F | 5′-GGTCGGTGTGAACGGATTTG-3′ |
|  | R | 5′-ATGAGCCCTTCCACAATG-3′ |
| *Ctsk* | F | 5′-CAGCTTCCCCAAGATGTGAT-3′ |
|  | R | 5′-GAAGCACCAACGAGAGGA-GA-3′ |
| *Acp5* | F | 5′-TCCTGGCTCAAAAAGCAGTT-3′ |
|  | R | 5′-ACATAGCCCACACCGTTCTC-3′ |
| *Nfatc1* | F | 5′-CACATTCTGGTCCATACGA-3′ |
|  | R | 5′-CGTGTAGCTGCACAATGG-3′ |
| *Oscar* | F | 5′-CTGCTGGTAACGGATCAGCTCCCCAGA-3′ |
|  | R | 5′-CCAAGGAGCCAGAACCTTCGAAACT-3′ |
| *Atp6v0d2* | F | 5′-CAGAGCTGTACTTCAATGTGGAC-3′ |
|  | R | 5′-AGGTCTCACACTGCACTAGGT-3′ |
